# Dissecting Immunosuppressive Cell Communication Patterns Reveals JunB Proto-Oncogene (JUNB) Shaping a Non-Inflamed Tumor Microenvironment

**DOI:** 10.3389/fgene.2022.883583

**Published:** 2022-06-24

**Authors:** Hualin Chen, Gang Chen

**Affiliations:** Department of Urology, The First Affiliated Hospital of Chongqing Medical University, Chongqing, China

**Keywords:** JunB, immunosuppressive, tumor microenvironment, cell communication, ScRNA-seq, molecular subtype

## Abstract

**Background:** Immunosuppressive cell interactions are responsible for tumor progression and metastasis, as well as anti-tumor immune dysfunction. However, the communication pattern remains unclear.

**Methods:** We first integrated two single-cell RNA-seq datasets (GSE72056 and GSE103322) of different tumor types to increase the diversity of immunosuppressive cells. Then, based on the analysis results of the communication network, gene regulatory network (GRN), and highly activated pathways, we identified the hub gene in the immunosuppressive tumor microenvironment (TME). To further explore the molecular features of the identified gene, we performed several *in silico* analysis and *in vitro* experiments including qRT-PCR and CCK-8 assay.

**Results:** Four types of immunosuppressive cells were identified, including cancer-associated fibroblasts (CAFs), tumor-associated macrophages (TAMs), tumor-associated neutrophils (TANs), and regulatory T cells (Tregs). Based on GRNs and the interactions of immunosuppressive cells and tumor cells, we constructed an intercellular communication signature that divided the pan-cancer TME into two clusters with distinct immunological features and different responses to immunotherapy. In combination with pathway analysis, JunB proto-oncogene (JUNB) was identified as the hub gene of the immunosuppressive TME, and it designed a non-inflamed TME of bladder cancer according to evidence that JUNB was negatively correlated with immunomodulators, chemokines, major histocompatibility complex molecules, immune cell infiltration abundances, anti-cancer immune response, and immune checkpoint inhibitors. Moreover, JUNB may predict an unfavorable response to immunotherapy. The signaling network of the four types of cells demonstrated the dominant roles of CAFs and TAMs in the TME. Further investigation uncovered that the complement signal was highly activated in the interactions between subpopulations of the inflammatory phenotype of CAFs and TAMs. Functional experiment results demonstrated the upregulated JUNB in bladder cancer tissues and low-immunity-score tissues. In addition, CAFs showed a pro-tumor proliferation effect *via* JUNB.

**Conclusion:** Our findings gave insights into the immunosuppressive TME communication network and provided potential therapeutic targets.

## Introduction

The tumor microenvironment (TME) consists of tumor cells, stromal cells, and immune cells, as well as non-cellular components such as chemokines. The striking advance in tumor biology demonstrates that the TME evolves with tumor development and progression, such as the dynamic changes in immunological characteristics. The formation of an immunosuppressive status of the TME is a hallmark of most malignancies. In the immunosuppressive TME, malignant cells inhibit the anti-tumor immune responses such as reducing anti-tumor T-cell activation, inhibiting T-cell proliferation, impairing T-cell survival through secretion of pro-tumor molecules, and colluding with tumor-associated non-malignant cells (Vinay et al., 2015; Labani-Motlagh et al., 2020). Therefore, it is imperative to uncover the molecular mechanisms within the immunosuppressive TME.

It is generally accepted that four cell types with immunosuppressive properties are responsible for TME re-programming, including cancer-associated fibroblasts (CAFs), tumor-associated macrophages (TAMs), tumor-associated neutrophils (TANs), and regulatory T cells (Tregs). As suggested by the literature, CAFs can release matrix metalloproteinases (MMPs) to promote tumor invasion and metastasis and exosomes to help tumor cells display the EMT phenotype (Bu et al., 2019). CAFs can also hinder anti-tumor immunity by secreting pro-inflammatory cytokines, as reported by Benyahia et al. (2017). Correlations between TAM infiltration level and unfavorable prognosis have been addressed in several malignancies (Zhang et al., 2012; Jung et al., 2015). TAMs of the immunosuppressive phenotype can support tumor progression, dissemination, angiogenesis, and immune suppression by excreting several types of molecules, such as MMPs, TGF-β, and vascular endothelial growth factor (VEGF) (Wang et al., 2021). TANs function an immunosuppressive role in the TME by promoting angiogenesis and metastasis and inhibiting effector CD8^+^ T-cells. TANs can also interact with other immunosuppressive cells like TAMs and Tregs to protect the tumor cells. Zhu et al. (2017) uncovered a strong correlation between the number of infiltrating TANs and poor survival (Zhu et al., 2017). Such significant findings also have been documented in Tregs (Kosaka et al., 2013). Collectively, these immunosuppressive cells and the molecules released contribute to the immunosuppressive features of the TME. They can induce tumor progression and attenuate the effective anti-tumor therapy. Therefore, deciphering the complex immunosuppressive cell communication pattern can help us understand the molecular mechanisms behind cancer cell progression and therapeutic resistance.

Recently, single-cell RNA-sequencing (scRNA-seq) made it increasingly possible to unveil the complex heterogeneity and intercellular communications in immunosuppressive TME with remarkable resolution. In this study, we depicted the communication patterns of the four immunosuppressive cells and tumor cells by scRNA-seq. Based on GRNs, a communication signature was constructed and showed remarkable performance in TME phenotype discrimination and immunotherapeutic response prediction in the bulk RNA-seq. Ultimately, we identified the hub gene JUNB that shaped a non-inflamed TME and predicted a negative immunotherapy response.

## Materials and Methods

### Data Acquisition

To depict the pan-cancer intracellular communication patterns within the immunosuppressive TME, we integrated two scRNA-seq datasets including a melanoma cohort (GSE72056) (Tirosh et al., 2016) and a head and neck squamous cell cancer (HNSC) cohort (GSE103322) (Puram et al., 2017).

Bulk RNA-seq data across 33 The Cancer Genome Atlas (TCGA) tumor types were downloaded from the UCSC Xena platform (https://xenabrowser.net/). Transcriptome data of 28 and 101 clinical tumor samples treated with immune checkpoint inhibitors (ICIs) were downloaded with the accession number GSE78220 (Hugo et al., 2016) and GSE91061 (Riaz et al., 2017), respectively.

### Single-Cell Analysis

Standard Seurat integration pipeline was utilized to analyze the two scRNA-seq datasets. To be specific, we first performed quality control to remove low-quality genes and cells, and then created potential anchors based on top variable genes. Subsequently, an integrated new matrix with 2,000 features across 10,547 cells was built by the IntegrateData function implemented in Seurat (Hao Y. et al., 2021). After dimensionality reduction, clustering, and manual annotation, four immunosuppressive cell types including CAFs, TAMs, Tregs, and TANs with two malignant cell types including melanoma and HNSC were identified and extracted for the following analysis.

Positive marker genes of each immunosuppressive cell type were identified by the FindMarker function and intersected with each by the VennDiagram package (Chen and Boutros, 2011). Ultimately, shared marker genes of each cell type were selected for the following analysis.

### GRNs Analysis, Cell–Cell Communication Analysis, and Gene Set Enrichment Analysis

Using scRNA-seq data, SCENIC maps TFs onto GRNs and integrates various cell types to infer cell-specific GRNs. There are two fast and efficient GRN inference algorithms, GRNBoost2 and GENIE3. Analytic procedures were carried out following the standard pipeline, namely 1) identification of target genes that are co-expressed with TFs utilizing GENIE3, 2) identification of regulons by cis-regulatory motif enrichment analysis using RcisTarget, and 3) scoring the activity of each regulon on single cell types by AUCell (Aibar et al., 2017; Hao Q. et al., 2021).

Based on the prior knowledge of signaling and GRNs, NicheNet can infer active ligands and their gene regulatory effects on interacting cells (Browaeys et al., 2020). LR pairs interaction analysis was conducted utilizing the CellChat with the constructed LR database: CellChatDB. Communication probability on the signaling pathway was assessed by summarizing the communication probabilities of corresponding LR pair interactions (Jin et al., 2021).

Pathway or gene set activity was scored by the GSVA package at the single-cell level (scRNA-seq) or bulk tumor level (TCGA pan-cancer) (Hänzelmann et al., 2013).

### Pseudo-Time Analysis

R package monocle was employed to perform the pseudo-time analysis (Trapnell et al., 2014). CellDataSet object was constructed by converting the Seurat object. The FindAllMarkers function in Seurat was employed to select ordering genes. After dimensional reduction and trajectory inference, gene translational changes were mapped to the pseudo-time by the plot_cell_trajectory function. Branches analysis in single-cell trajectories was carried out by a special statistical test: branched expression analysis modeling (BEAM).

### Clinical Samples

Human bladder cancer tissues and paired paracancerous tissues were obtained from ten patients receiving partial/radical cystectomy at The First Affiliated Hospital of Chongqing Medical University (CQMU) from April 2021 to May 2021, under the permission of the Ethics Committee of The First Affiliated Hospital of CQMU (Approval Number: 2020-155). The exclusion criteria were: 1) patients who had received previous bladder cancer treatment including intravesical/systemic chemotherapy, radiotherapy, immunotherapy, and targeted therapy, 2) non-muscle invasive bladder cancers, and 3) recurrent and metastatic tumors. Surgically resected clinical samples were stored in liquid nitrogen for the following experiments. We obtained written informed consent from each patient and conducted the study according to the Helsinki Declaration.

### Cell Culture

We obtained human bladder cancer cell lines T24 from the Chinese Academy of Sciences (Shanghai, China) and cultured them according to previous descriptions. CAFs were isolated from adjacent bladder tumor tissues and cultured as described previously (Dong et al., 2021). In brief, tumor tissues were cut into small pieces (1 × 1 × 1 mm^2^) and washed with phosphate buffered saline solution (PBS, Thermo Fisher Scientific, Waltham, MA) three times. Then, small pieces were digested with 25 μg/ml hyaluronidase (Thermo Fisher Scientific, Waltham, MA) and 160 μg/ml collagenase I (Thermo Fisher Scientific, Waltham, MA) for 2 h at 37°C. Small pieces were washed with the medium and then cultured in Dulbecco’s Modified Eagle’s Medium (DMEM) (Sigma, United States) at a 37°C incubator with 5% CO_2_. 10% fetal bovine serum (FBS) (Thermo Fisher Scientific, Waltham, MA) and 100 U/ml streptomycin/penicillin were supplemented into the medium.

### siRNA Transfection and Transwell Co-Culture

JUNB siRNA (Arcturus Therapeutics, Inc.) was transfected with OPTI-MEM (MediaTech, United States) following the manufacturer’s protocols. The siRNA sequence was as follows: FLI-1-specific siRNA: 5′- GTC​TCT​AAA​GAG​TTT​ATT​TTA​AG-3’.

As for the 0.4 µm transwell coculture system (Corning, Glendale, AZ), CAFs (upper chamber) were indirectly cocultured with T24 cells (lower chamber). The experimental groups were as follows: control (T24 cells), siJUNB-coculture (siJUNB CAFs cocultured with T24 cells), and wild-type coculture (wild-type CAFs cocultured with T24 cells).

### Cell Viability

Cell viability assay was conducted by Cell Counting Kit-8 (CCK-8, Beyotime, China) according to the manufacturer’s instructions. In brief, T24 cells were seeded into 96-well plates with 100 μl culture medium from CAFs for 48 h. Then, the medium of each well was replaced by 10 μl of CCK-8 solution and the cells were incubated at 37°C in 5% CO_2_ for 1 h. The absorbance was measured at 490 nm.

### RNA Extraction and Quantitative Real-Time PCR

Total RNA was obtained from cultured bladder tumor cells, CAFs, and human tissues with a total RNA extraction kit of UNIQ-10 column Trizol type (Sangon Biotech, China) following the instruction of the manufacturer. Reverse transcription was subsequently performed utilizing the RR047 cDNA synthesis kit (Thermo Fisher Scientific, Waltham, MA) and qRT-PCR was performed in a 7300 Plus Real-Time PCR System (Thermo Fisher Scientific, Waltham, MA) using the Phusion U Green Multiplex PCR Master Mix (Invitrogen, United States). The mRNA expression of genes was normalized to the levels of GAPDH expression. The sequences of primers were as follows: JUNB-F:5′-ACAGTACTTTTACCCCCGCG-3′, JUNB-R: 5′-TGA​GCG​TCT​TCA​CCT​TGT​CC-3’.

### Statistical Analysis

Correlation analysis between variables was performed by Spearman or Pearson coefficients. Continuous variables between binary groups were compared by the *t*-test or Mann–Whitney *U* test if they failed to fit a normal distribution. Survival analyses were conducted utilizing the Kaplan-Meier method with the log-rank test. The significant level *p* value was set as 0.05. All statistical analyses and data visualization were performed in R (version 3.6.3).

## Results

### Shared Positive Markers of Immunosuppressive Cells

**FIGURE 1:**
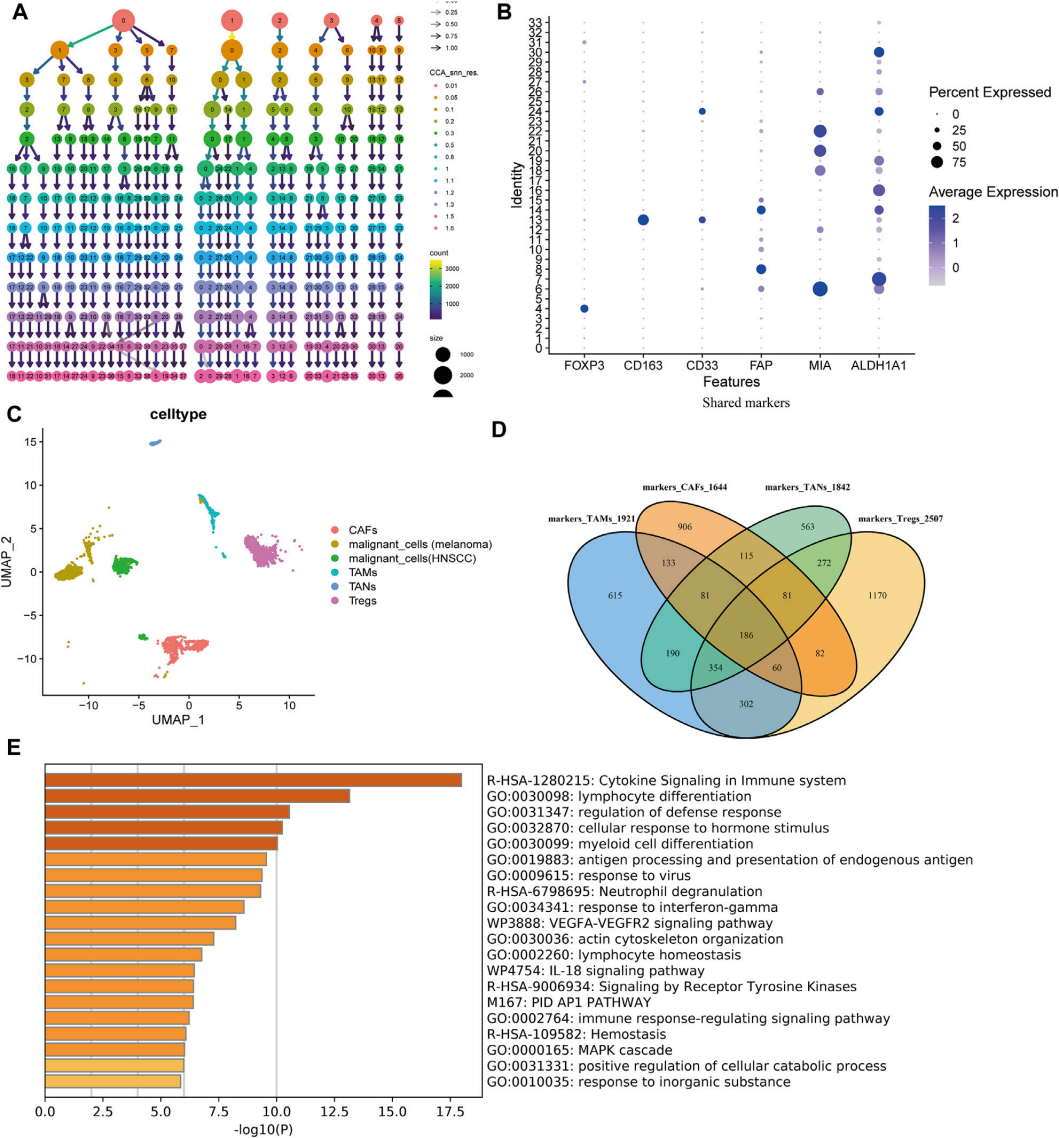
Cell type annotation and positive marker identification. **(A)** Optimal resolution deciding dendrogram. Under the resolution of 1.3, all cells achieved the optimal clustering and no mixed cells in the same cluster. **(B)** Cell type-specific markers. **(C)** Clustering and annotating immunosuppressive cells (CAFs, TAMs, Tregs, and TANs) and malignant cells (melanoma and HNSCC). **(D)** 186 shared positive markers of each immunosuppressive cell. **(E)** The top 20 enriched terms across the 186 shared markers.

A total of 34 clusters were identified under the resolution of 1.3 (Figure 1A). Previously documented markers including FOXP3 (Tregs), CD163 (TAMs), CD33 (TANs), FAP (CAFs), MIA (melanoma), and ALDH1A1 (HNSC), were used for cell-type annotation (Figure 1B). Next, these well-annotated cells were extracted for further analysis (Figure 1C). After the positive marker genes of each immunosuppressive cell were identified, the 186 shared genes were set as the universe immunosuppressive marker genes by VennDiagram (Figure 1D). Enrichment analysis demonstrated that several immune-related pathways were enriched by the 186 marker genes (Figure 1E).

### Highly Activated JUNB Within the TME

GRNs were defined as intricate networks composed of transcription factors (TFs) and their downstream targeted genes and functioned as cell states determination and maintenance. Single-cell regulatory network inference and clustering (SCENIC) analysis was performed to reconstruct GRNs from scRNA-seq and score the activity of regulons (a TF together with its target genes comprises a regulon) in each cell to identify the recurrent cellular states.

**FIGURE 2:**
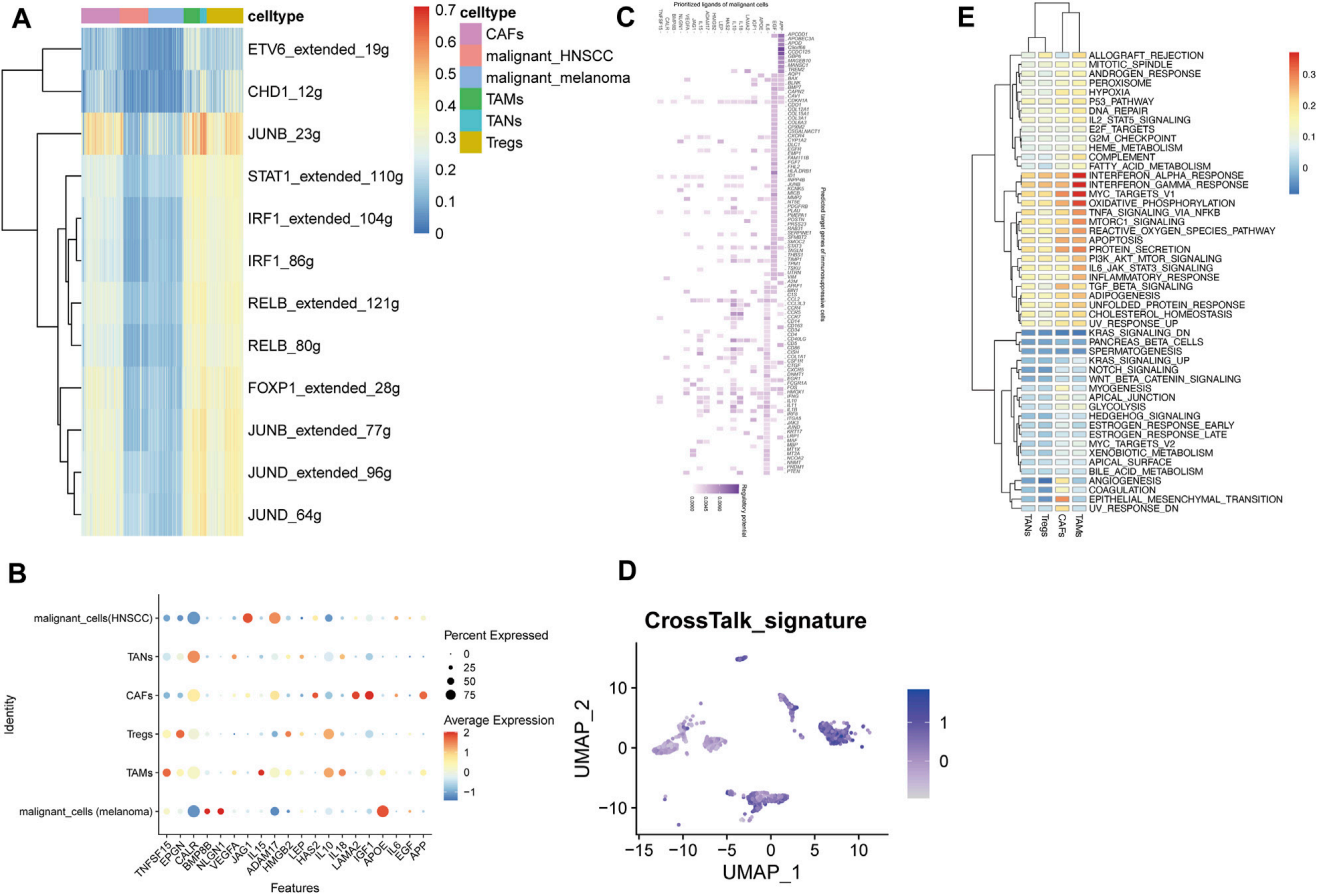
GRNs analysis and communication network of immunosuppressive cells and malignant cells. **(A)** Regulon activity across immunosuppressive and malignant cells. **(B)** The top 20 ligands among immunosuppressive and malignant cells. **(C)** The Ligand-target gene matrix denoted the top targeted genes of immunosuppressive cells by the top 20 ligands of malignant cells. **(D)** Activity score of the constructed communication signature. **(E)** Differences in pathway activity in four immunosuppressive cell clusters.

Based on the scRNA-seq dataset of 186 shared marker genes, ten regulons, specifically, JUNB, displayed the highest activity in immunosuppressive cells (Figure 2A). It has been well-documented that JUNB participates in tumorigenesis, progression, and invasion of several tumors like renal cell carcinoma and hepatocellular carcinoma as well as inhibition of the proliferation of adaptive immune cells (Szremska et al., 2003; Guo et al., 2009; Kanno et al., 2012). These results suggest that JUNB may play an essential role in the immunosuppressive TME.

To further dissect the immunosuppressive TME, TFs and their downstream-regulated genes were extracted for subsequent analysis.

### An Immunosuppressive TME Signature

Enormous evidence supported that the interactions between tumor cells and non-malignant cells may promote tumor growth and progression (Garner and de Visser, 2020; Bayik and Lathia, 2021). To depict the crosstalk pattern in the immunosuppressive TME, NicheNet was utilized to model the molecular interactions by analyzing the expression profiles of ligand-receptor (LR) pairs and their regulatory genes between tumor cells and immunosuppressive cells.

Remarkably, most of the 20 prioritized ligands such as APP, EGF, and IL6, were associated with the process of tumor formation, progression, and invasion as well as confronting the anti-tumor immune response (Figures 2B,C) (Mendelsohn and Baselga, 2000; Lim et al., 2014; Kumari et al., 2016; Shanmugalingam et al., 2016; Liang et al., 2020).

Subsequently, an immunosuppressive TME gene signature was constructed based on the 14 overlapped genes of regulons and targeted genes. The expression pattern of the signature was upregulated in immunosuppressive cells compared to malignant cells (Figure 2D).

### Molecular Subtypes of the Pan-Cancer TME Determined by the Signature

**FIGURE 3:**
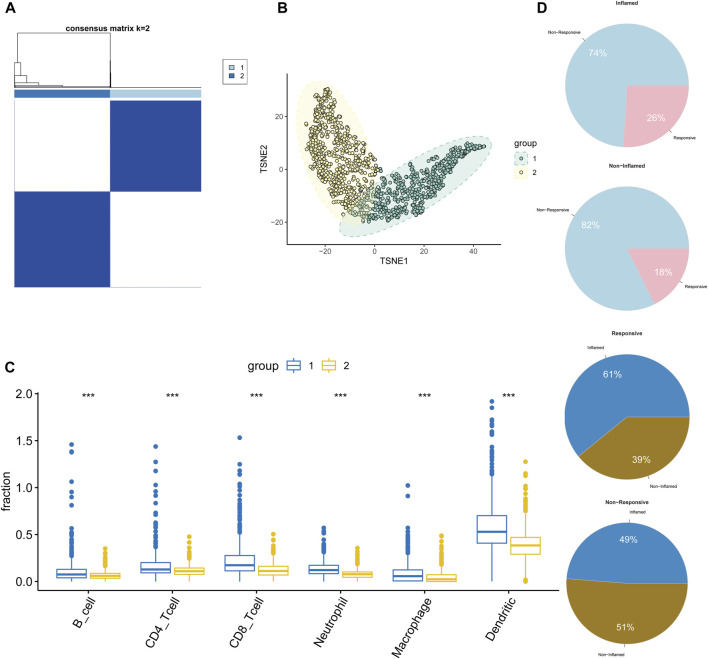
Distinct molecular features of pan-cancer TME revealed by communication signature. **(A)** Two groups of TCGA pan-cancer by consensus clustering. **(B)** A t-SNE plot indicated remarkable separations. **(C,D)** The differences in infiltration levels of immune cells **(C)** and immunotherapy response **(D)** in two groups.

To further explore the molecular mechanisms of the constructed signature, we analyzed the transcriptome dataset of TCGA pan-cancer by unsupervised consensus clustering (Wilkerson and Hayes, 2010). Tumor samples were categorized into two groups under the optimal separation (Figure 3A). T-distributed stochastic neighbor embedding (t-SNE) results showed perfect separation quality (Figure 3B).

Subsequently, to unveil the molecular features of two groups at the TME level, the infiltration abundances of six immune cells including B cells, CD4/8 T-cells, neutrophil, macrophage, and dendritic cells were inferred using the online tool TIMER (Li et al., 2017). Tumors in group one showed higher infiltration levels of both innate and adaptive immune cells than those in group two (Figure 3C). Results were validated by the webtool xCell (Supplementary Figure S1A) (Aran et al., 2017). Therefore, the TME phenotypes of groups one and two were considered non-inflamed and inflamed, respectively. Two independent datasets (GSE78220 and GSE91061) were used to reproduce and validate the results. As expected, two groups of tumors with distinct immune infiltration phenotypes were identified (Supplementary Figure S1B, C). Collectively, the signature can shape the TME into two groups with distinct immune infiltration characteristics and extents.

As documented in previous studies, immunotherapeutic response varied dramatically between inflamed and non-inflamed TME (Trujillo et al., 2018). Considering the immune inhibitive and immunotherapeutic resistant features of immunosuppressive TME, we investigated the association between TME phenotypes (inflamed and non-inflamed) and the response to immunotherapy (response: CR/PR and non-response: PD/SD) by exploring the GSE91061 dataset which stored the transcriptome data of melanoma after ICIs treatment. Compared to the non-inflamed tumor, the inflamed had a higher immunotherapeutic response rate (26% vs. 18%) and the responsive phenotype favored inflamed tumors compared to non-inflamed (61% vs. 49%) (Figure 3D).

### JUNB Was Identified as the Hub Gene

Pathway analysis with GSVA revealed that INF-α/γ response, MYC targets, and oxidative phosphorylation were exclusively enriched in TAMs. While angiogenesis, coagulation, and epithelial-mesenchymal transition (EMT) were specifically upregulated in CAFs, suggesting the possible relationship between CAFs activation and tumor metastasis (Figure 2E) (Mittal, 2018). Among the top activated pathways, 9/14 (64%) signature genes including JUNB, DUSP1, ID2, KLF6, NFKBIA, NR4A2, PFKFB3, TNFAIP3, and ZFP36, were presented in the TNF α/NF-κB pathway which has been widely reported in tumor cell invasion and metastasis promoting and drug resistance (Wu and Zhou, 2010). Together with GRNs analysis, the JUNB was selected for further analysis.

### JUNB Shaped a Non-Inflamed TME of Bladder Cancer

Considering the essential roles of JUNB revealed by scRNA-seq and the immunological feature of bladder cancer, we decided to explore the extensive molecular mechanisms in this specific cancer.

**FIGURE 4:**
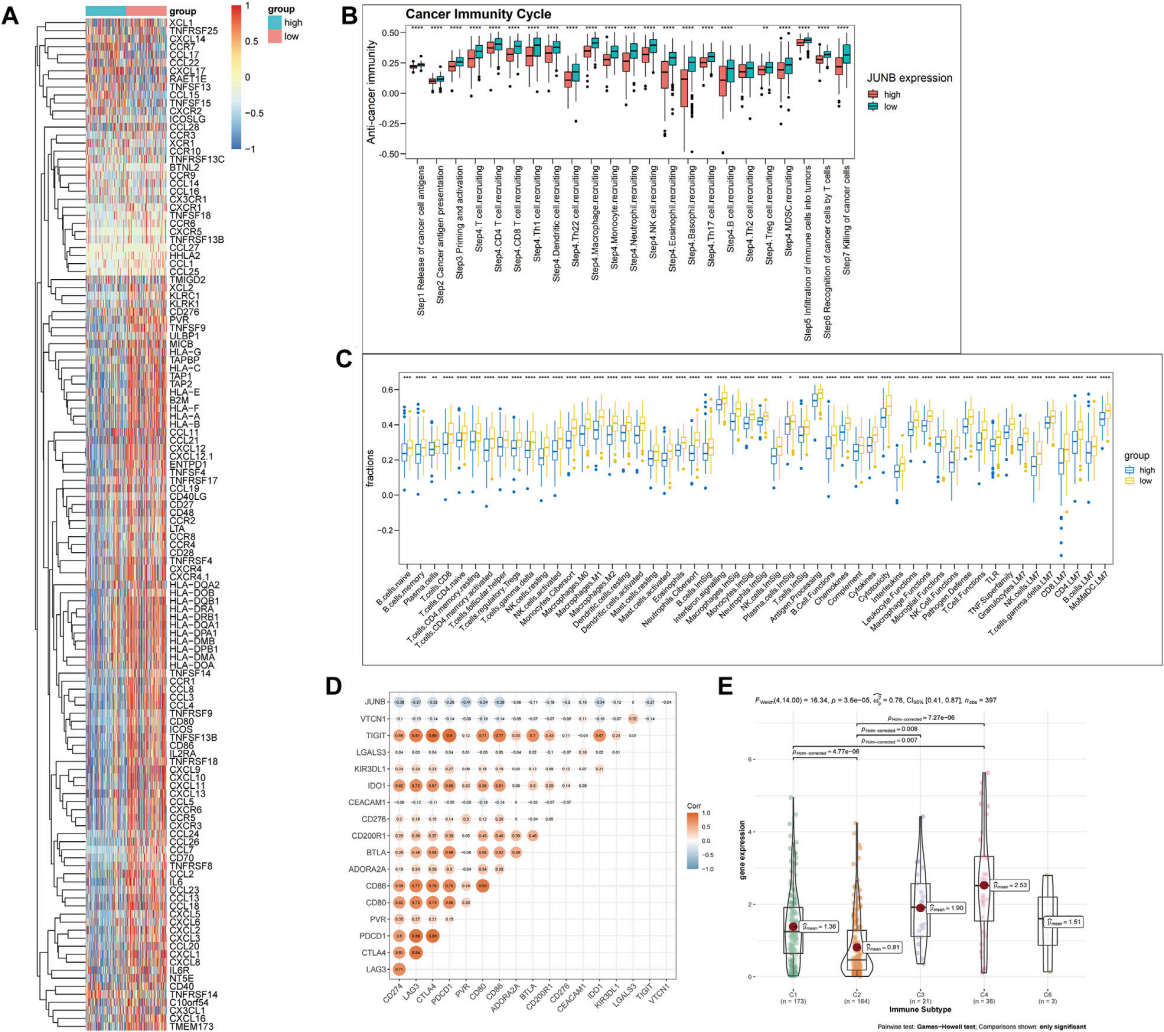
JUNB shaped a non-inflamed TME and predicted a non-sensitive response to ICIs treatment. **(A)** Expression of immunostimulators, chemokines, MHC molecules and receptors, **(B)** differences in anti-cancer immunity cycle, and **(C)** differences in infiltration levels of immune cells between high- and low-JUNB groups. **(D)** Correlation between the expression of JUNB and immune checkpoint inhibitors. **(E)** The expression of JUNB across immune subtypes.

First, we analyzed the correlation between the expression profiles of JUNB and immunostimulators, chemokines, and MHC molecules*.* Significantly negative correlations were found in most immunomodulators and MHC molecules. JUNB was significantly negatively correlated with two vital chemokines (CXCL9 and CXCL10) which functioned as recruiting CD8^+^ T-cells into the TME. Similar results were also found in chemokines like CXCL11, CCL21, CCL4, CCL3, and CCL2 and paired receptors including CXCR3, CCR6, and CCR1. These chemokines can induce the recruitment of effector tumor-infiltrating immune cells such as antigen-presenting cells, Th17 cells, and CD8^+^ T-cells (Figure 4A).

Next, we explored the feature of JUNB in anti-cancer immune responses in which a series of progressive events were required to be initiated and enabled to proceed and amplify iteratively. Strikingly, activities of all steps in the cancer immunity cycle were downregulated in the high-JUNB group (Figure 4B).

We obtained one curated immune signature gene set combined from four sources (leukocyte signature matrix 22 or LM22, leukocyte signature matrix seven or LM7, immune cell gene signature or ImSig, and the NanoString immune signature panel (https://www.nanostring.com)) from one previous study (Das et al., 2020) and scored the signature utilizing GSVA package. In line with previous results, JUNB was downregulated in tumors with high infiltration levels of various adaptive and innate immune cells (Figure 4C).

Evidence has reported the negative correlation between non-inflamed TME and the expression of immune checkpoints. Consistently, JUNB showed negative correlations with most immune checkpoints including CD247, LAG3, CTLA4, and PD-1(PDCD1) (Figure 4D).

Thorsson et al. identified six immune subtypes (C1: wound healing, C2: IFN-*γ* dominant, C3: inflammatory, C4: lymphocyte depleted, C5: immunologically quiet, and C6: TGF-*β* dominant) across TCGA pan-cancer and found that distinct subtypes characterized diverse TME (Thorsson et al., 2018). As expected, JUNB was significantly upregulated in the C4 subtype which exhibited a prominent M2 macrophage response (Figure 4E).

Collectively, JUNB demonstrated strong and significant correlations with the development of a non-inflamed TME.

### JUNB Predicted an Unfavorable Response to ICI Treatment

Theoretically, upregulated JUNB suggested a low response rate to ICIs due to the non-inflamed TME defined. Ayers and colleagues defined a pan-cancer T-cell-inflamed gene expression profile (GEP) that can predict the therapeutic response of ICIs (Ayers et al., 2017). In the study, JUNB was negatively correlated with the T-cell-inflamed GEP (R = 0.37, *p* < 0.001) (Supplementary Figure S2A). Another concern related to the ICIs treatment was the adverse effect of hyperprogressive disease with an incidence ranging from 4% to 29% according to the previous studies (Adashek et al., 2020). Hence, we explored the correlation between JUNB and hyperprogression-related positive markers including FGF3, FGF4, FGF19, MDM2, MDM4, and DNMT3A, and negative markers such as CDKN2A and CDKN2B. As expected, high expression of JUNB was positively correlated with MDM4 and negatively correlated with CDKN2B, indicating the higher incidence of ICIs-induced hyperprogression in the high-JUNB group (Supplementary Figure S2B).

### CAFs and TAMs Were Identified as the Core

**FIGURE 5:**
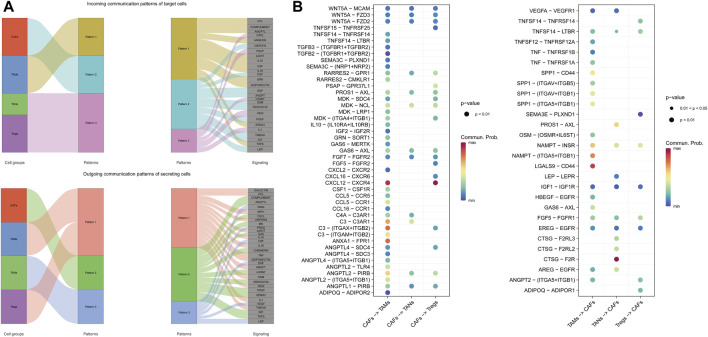
The intracellular communication network of immunosuppressive cells. **(A)** Incoming (upper panel) and outgoing (lower panel) communication patterns of target and secreting cells, respectively. **(B)** All significant LR interactions between CAFs and the immunosuppressive cells.

To delineate the crosstalk pattern within immunosuppressive cells, we analyzed the intercellular signaling pathways by CellChat and clustered all significant incoming and outgoing signals into three patterns (Figure 5A). Within the three incoming patterns, signals targeting TAMs and CAFs were identified as pattern one and pattern two, respectively. While pattern three consisted of signals from two cell types: TANs and Tregs. Remarkably, pattern one and pattern two accounted for the majority of all incoming signals.

When functioning as signal senders, TAMs and Tregs were set as one cluster from which signals were clustered into pattern one. Signals sent by CAFs and TANs were identified as pattern two and pattern three, respectively. Similarly, a large number of outgoing signals have been clustered into pattern one and pattern two. Interestingly, only the signals either targeting at or sending from CAFs exclusively formed one pattern, and signals related to CAFs and TAMs accounted for the majority of the intercellular communication networks, indicating the critical roles of CAFs and TAMs in the immunosuppressive TME.

Next, we investigated all significant signals related to CAFs at the LR pairs level and noticed the exceedingly frequent communication between CAFs and TAMs (Figure 5B). Accumulated evidence indicated the critical roles of the CXCL12 − CXCR4 axis in tumor proliferation, progression, vascularization, and migration as well as the formation of an immunosuppressive TME by the exclusion of immunoreactive cells like T-cells. And the axis targeting therapeutic efficiency has been widely proved in multiple cancers, such as metastatic breast cancer, ovarian cancer, and glioblastoma (Chen et al., 2019; Santagata et al., 2021). In our study, this pro-tumorigenic axis was highly activated in the immunosuppressive TME, further suggesting that the targeting therapy may rescue the compromised anti-tumor immune activity. Therefore, CAFs and TAMs were considered the core within the TME, and CAFs tended to serve as senders.

### Subpopulation Identification and Pseudo-Time Analysis

**FIGURE 6:**
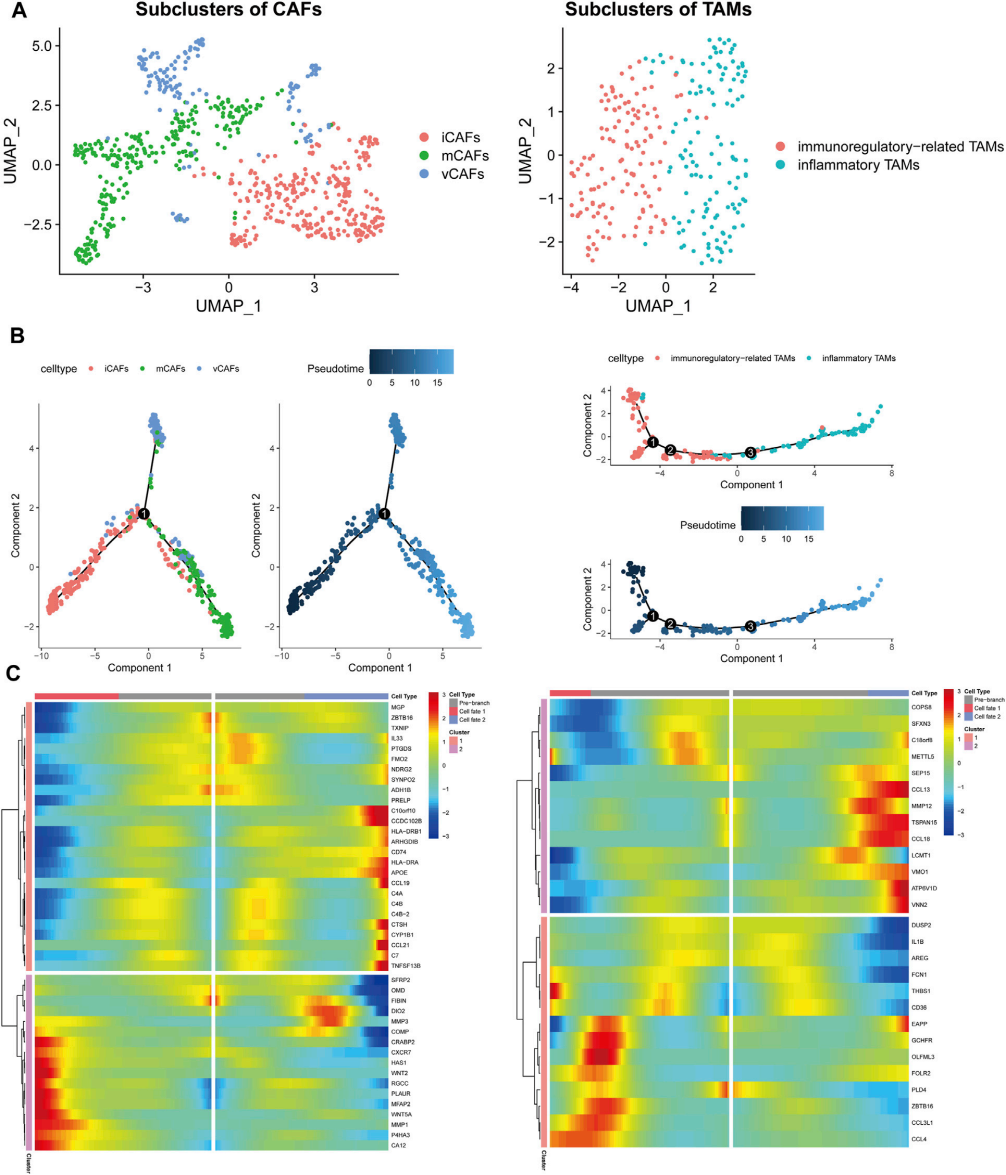
Subpopulations of CAFs (left panel) and TAMs (right panel) identification and trajectory development investigation. **(A)** Further clustering CAFs and TAMs according to reported phenotype markers. **(B)** Investigation differentiation trajectories of subpopulations of CAFs and TAMs through pseudotime analysis. **(C)** Trajectory branches analysis denoted cell-fate dependent genes.

CAFs were re-clustered and annotated as vascular CAFs (vCAFs), matrix CAFs (mCAFs), and inflammatory CAFs (iCAFs) by markers reported by Zhang et al. (2020). Subpopulations of immunoregulatory-related and inflammatory TAMs were also identified based on markers from one previous study (Figure 6A) (Wang et al., 2021).

To explore the trajectory development, pseudo-time analysis was then performed on the subpopulations of each cell type. iCAFs were projected onto one root with two branches corresponding to mCAFs and vCAFs, respectively. Consistent with the previous study, immunoregulatory-related TAMs were projected onto the root (Figure 6B) (Wang et al., 2021). Single-cell branch analysis was then conducted to identify cell differentiation-dependent genes (Figure 6C).

### In-Depth Intercellular Crosstalk Analysis

**FIGURE 7:**
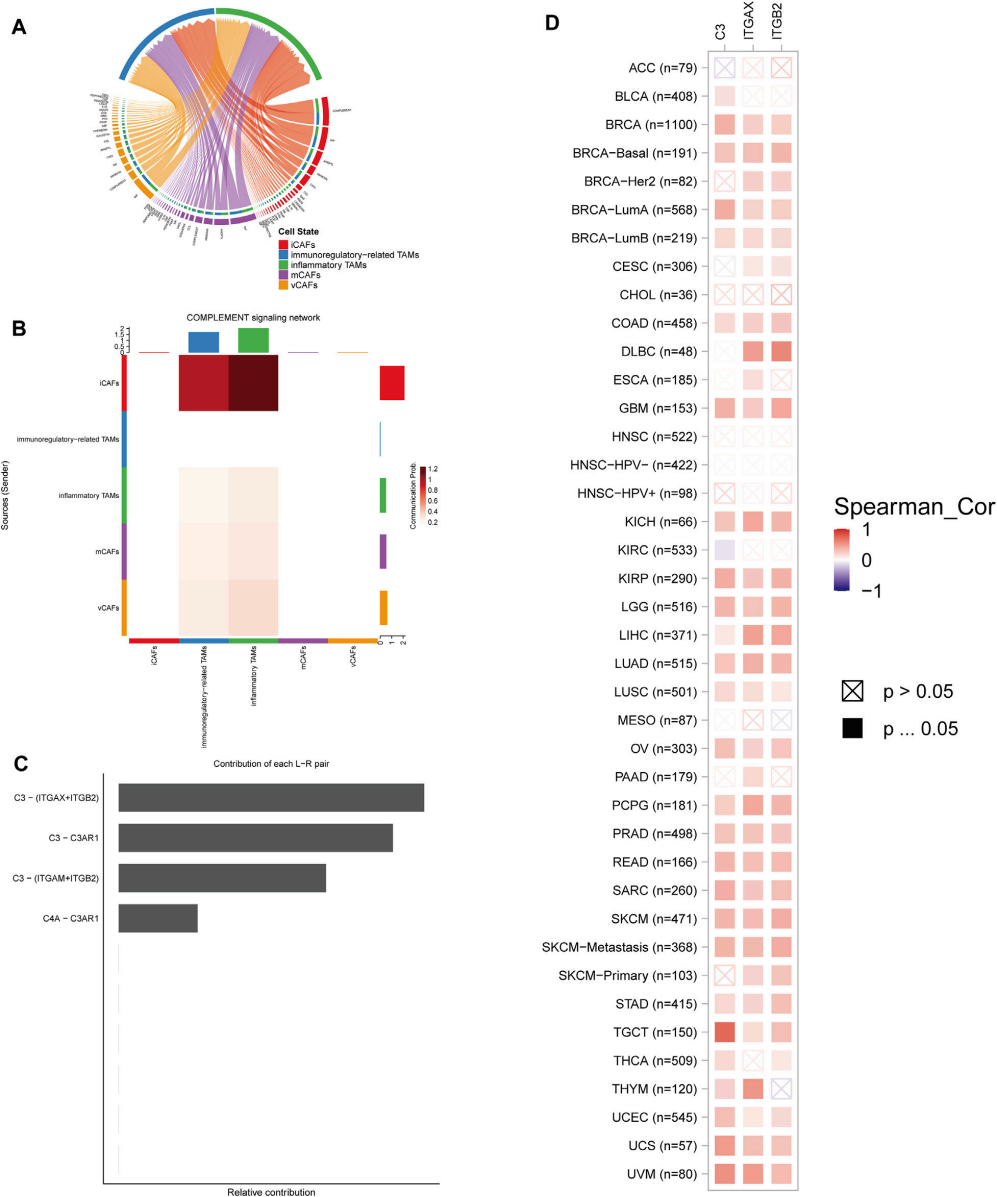
The complement signal network was highly activated between iCAFs and inflammatory TAMs. **(A)** Communication signals sent from CAFs and targeting TAMs. **(B)** Complement signaling network was highly activated between the inflammatory phenotypes of CAFs and TAMs. **(C)** Contribution of each LR pair of complement signal to the network. **(D)** Pan-cancer correlation between the expression of JUNB and C3-(ITGAX + ITGB2).

The top two outgoing signals from CAFs were complement (dominant by iCAFs) and mif (dominant by vCAFs and mCAFs) (Figure 7A). Signaling network analysis indicated that the complement signal was highly activated between the inflammatory phenotype of CAFs (iCAFs) and TAMs (inflammatory TAMs) (Figure 7B). Within the complement signal, complement component C3 and receptors showed cardinal communicational contributions (Figure 7C). Subsequently, we utilized webtool TIMER (version 2.0) to perform the correlation analysis and unveiled positive correlations between JUNB and the LR pair in a majority of cancer types (Figure 7D).

Considering the manifold and intricate functions of the complement system, the correlation between JUNB and individual LR pairs was not sufficient to clarify the complement characters in the TME, to be specific, which activity dominated remained unclear: anti-tumor or pro-tumor?

### Functional Assay

As one of the most immunogenic neoplasm, bladder cancer is a promising target for immunotherapy such as ICIs and intravesical BCG. Although multiple trials have demonstrated the effective roles of ICIs, fewer than half of advanced bladder cancers get benefit from the treatment, suggesting significant individual heterogeneity. Previous studies have addressed the roles of CAFs in immunotherapeutic resistance in bladder cancer. However, the molecular mechanisms remained unclear.

**FIGURE 8:**
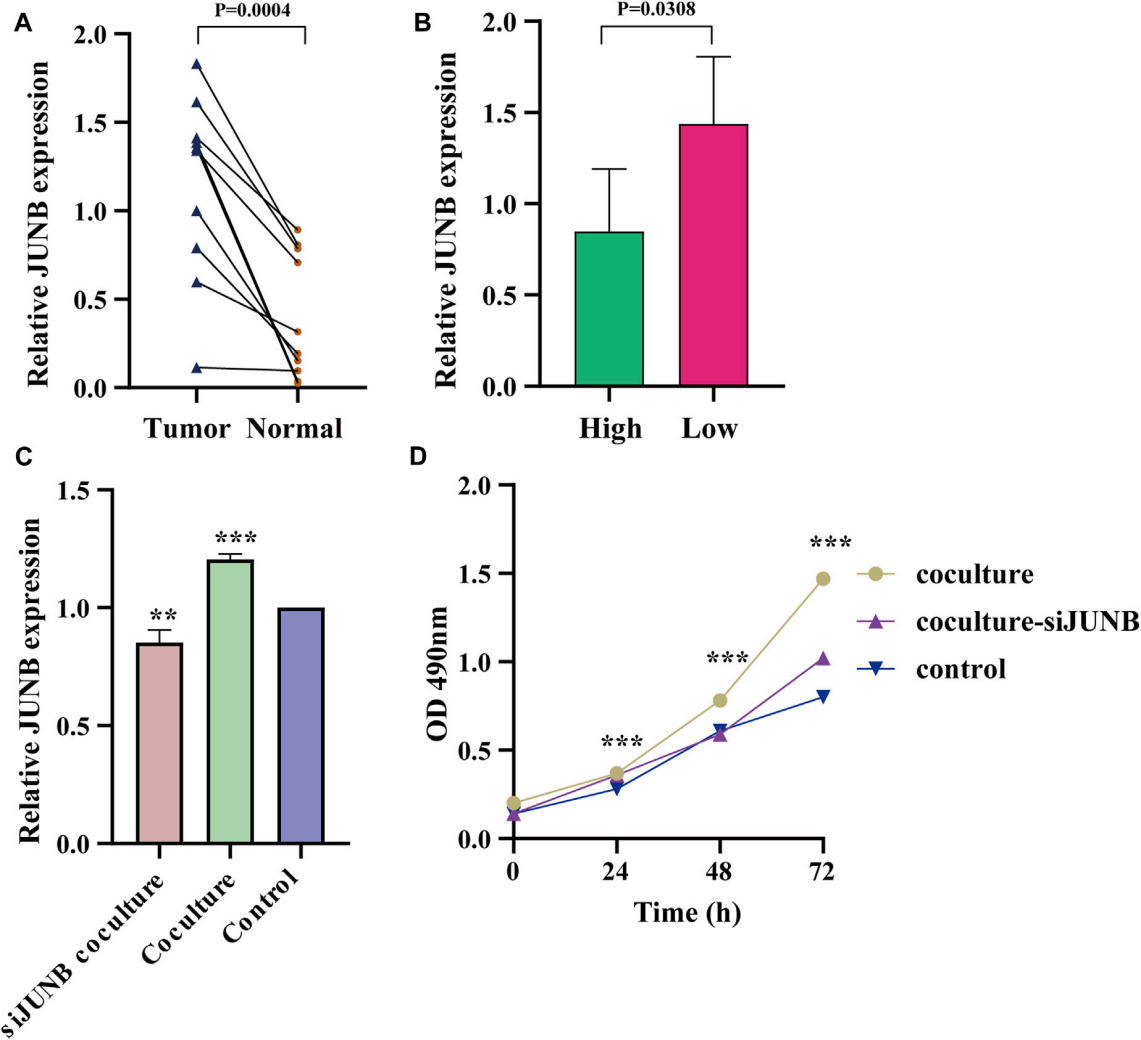
CAFs promoted bladder tumor proliferation by JUNB. **(A,B)** The expression of JUNB between bladder tumor and adjacent normal tissues **(A)**, and high- and low-immunity group **(B)**, by qRT-PCR analysis. **(C)** The expression of JUNB after JUNB knockdown, under co-culture conditions. **(D)** T24 cell viability when co-cultured with CAFs.

Hence, in the study, we analyzed the molecular characteristics of the gene JUNB in ten muscle-invasive bladder tumors. The results suggested that JUNB was upregulated in tumor tissues and the low immunity score group (Figures 8A,B). Moreover, CAFs showed a pro-tumor proliferation effect via JUNB as suggested by the CCK-8 assay (Figures 8C,D).

## Discussion

Tumor immunoresistance and immune escape are responsible for cancer progression and formation of the immunosuppressive and pre-metastatic niche, by modulating the recruitment and expansion of immunosuppressive cell populations such as CAFs, TAMs, Tregs, and TANs, and transforming the normal immune cells and stromal cells from a potentially anti-tumor state to a pro-tumor state in the aspects of phenotype and function. Immunosuppressive cells are essential components of TME in promoting tumor immune escape by compromising anti-tumor immunity and fascinating tumor metastasis via different mechanisms including cell-cell communication networks and GRNs (Liu and Cao, 2016; Wang et al., 2021). Therefore, dissecting the communication pattern of the immunosuppressive TME and identifying optimal therapeutic targets are needed in the immunotherapeutic revolutionized era.

In the study, we first identified four types of immunosuppressive cells and then constructed GRNs and communication networks. Together with putative genes, JUNB and JUND, two subunits of activator protein 1 (AP-1) TF, are the most highly activated regulons in the GRNs, indicating the significantly upregulated sub-network activity of the immunosuppressive cells. Increasing evidence shows that AP-1 functions as diverse and critical roles in the immune system such as T-cell anergy and exhaustion. By binding on the locus of the FOXP3 gene, AP-1 can fascinate the expression of this specific marker of Tregs. Therefore, we may speculate the AP-1 complexes' selective depletion can reinforce the responses of effective anti-tumor T-cells and amplify the efficacy of immunotherapy by removing the immune inhibition and damaging the immunosuppressive functions of Tregs.

Pan-cancer TME were categorized into two groups: the inflamed and the non-inflamed by the signature, and CD4/8 + T-cells tended to infiltrate the inflamed. It has been generally accepted that the inflamed TME has high infiltration abundance of lymphocytes and predicts an effective response to immunotherapy. As reported by Tumeh et al., inflammation positively correlated with ICIs treatment response and favored ICIs treatment response was evident in an inflammatory TME (Tumeh et al., 2014). Contrarily, the non-inflamed phenotype has “cold” tumor features including paucity of CD8^+^ T-cells and infiltration of immunosuppressive cells as well as inhibition of normal immune cells, and shows a poor response to ICIs treatment. To address this situation, expectation sets of ideas (e.g., epigenetic drugs, toll-like receptor agonists, and CD8^+^ T-cell receptor engineered T-cells) have been proposed to switch a non-inflamed TME into the inflamed one to augment the therapeutic response and improve overall survival (Morgan et al., 2006).

The M1/M2 macrophage polarization results from a response to TME cell–cell communication signals. Two phenotypes of macrophages are characterized by distinct inflammatory profiles: pro-inflammatory of M1 and anti-inflammatory of M2 (Orecchioni et al., 2019). The former presents the effective anti-tumor immunity, while the latter promotes tumor growth and serves an immunosuppressive role. INF-α (type I) and INF-γ (type II), in combination with toll-like receptor (TLR) agonists, are expected to activate anti-tumor macrophages, indicating a novel strategy of cancer immunotherapy (Müller et al., 2018). c-MYC is critical in the M2 polarization of macrophages and its target genes are closely associated with differentiation. Moreover, NF-κB signaling was upregulated in the TAMs and maintained the immunosuppressive phenotype. Once the signaling is inhibited specifically, TAMs switch to the M1 phenotype and function as tumor cell cytotoxic roles. Therefore, c-MYC and NF-κB signaling may be potential targets to “re-educate” the M1 macrophage phenotype, indicating a novel therapeutic approach in the era of specific target cell therapy (Hagemann et al., 2008). It has been well-documented that CAFs, in combination with secreted cytokines, act as a key player in the tumor EMT process (Goulet et al., 2019). One study by Katanov and Lerrer et al. explored the interaction networks of breast cancer and implied that NF-κB may be set as a target for CAFs inhibition to control tumor promotion (Katanov et al., 2015). These previous works provided a basic framework for the following research to explore preciously targeting therapy.

In the study, JUNB shaped a non-inflamed TME based on the negative correlations with the immunological status of TME. Downregulated expression of JUNB was significantly positively correlated with the majority of effective immune cell tracking chemokines and MHC molecules that participated in antigen-presenting. Correspondingly, various adaptive and innate immune cells were recruited and highly infiltrated in TME with low-expressed JUNB, and the cancer antigen-presenting step in stepwise anti-cancer immune response was also significantly activated. The upregulated expression of the gene in the C4 subtype also supported the unexpected findings. As commented earlier, a non-inflamed TME may undermine the immunotherapeutic response and contribute to an unfavorable prognosis of tumor patients who have received ICIs treatment. Furthermore, correlation analysis of T-cell inflamed and hyperprogression predicted the harmful effect of ICIs treatment in bladder cancer with upregulated JUNB. Further clinical pharmaceutical experiments are needed to clarify these pilot findings and develop specific therapeutic strategies based on the inflammation phenotype.

As the most abundant and crucial players of the TME, CAFs, and TAMs are in reciprocal and dynamic communication with the malignant cells to promote development and progression. In addition to interacting with malignant cells via cell-cell contact or soluble factors excretion, CAFs also play important roles in the crosstalk with immune cells including TAMs by secreting redundant soluble mediators such as cytokines and chemokines to therefore regulate immunity and sculpt the TME. Ample evidence supports the roles of CAFs in monocyte recruitment by LR pair interaction such as the CXCL12−CXCR4 axis, and pro-tumoral M2 phenotype differentiation (Monteran and Erez, 2019). Reciprocally, the M2 TAMs can regulate the activation of CAFs to promote the tumor progression and mediate the mesenchymal–mesenchymal transition (MMT) of CAFs to enhance the reactivity. Furthermore, activated CAFs participate in the TAMs phenotype swift from M1 type to M2 type.

Dissecting the interactive signaling network followed by subpopulations identification of CAFs and TAMs disclosed that the complement signal was predominantly upregulated between the inflammatory phenotype of 2 cell types, suggesting the crucial role of the signal within inflammatory TME. C3/CR3 (CD11b/CD18) is critical in regulating inflammation by mediating leukocyte migration and enhancing inflammatory mediators’ excretion (Fan and Edgington, 1993). Our findings enable molecular insights from the LR interaction level into the inflammatory single-cell phenotype and provide a fundamental framework for promising selective phenotype CAFs/TAMs-targeting therapy options.

Due to the limitation in sequencing depth of these two analyzed datasets, we can only identify four types of immunosuppressive cells from the TME, except for myeloid-derived suppressor cells (MDSCs) which also contribute to the immunosuppressive features of the TME (Wu et al., 2021). With more advanced scRNA-seq techniques and higher sequencing depth, more molecular traits within the immunosuppressive TME will be discovered in the following research.

## Conclusion

In conclusion, we identified four types of immunosuppressive cells, including CAFs, TAMs, TANs, and Tregs, within the integrated scRNA-seq datasets. Through an intercellular communication network, GRNs, and pathway analysis, we constructed one cell-cell interaction signature which categorized the pan-cancer into two clusters with distinct immunological status and different responses to ICIs treatment. As a hub gene in the communication network, JUNB shaped a non-inflamed TME and predicted a non-sensitive response to ICIs, serving as a promising target for therapy. Functional experiment results supported these findings and implied the important role of JUNB in the pro-tumor proliferation effect of CAFs.

## Data Availability

The original contributions presented in the study are included in the article/Supplementary Material; further inquiries can be directed to the corresponding author.

## Abbreviations

ACT, adoptive cell therapy; CQMU, Chongqing Medical University; CAFs, cancer-associated fibroblasts; EMT, epithelial–mesenchymal transition; FDA, Food and Drug Administration; GEP, gene expression profile; GSVA, gene set variation analysis; GRNs, gene regulatory networks; HNSCC, head and neck squamous cell carcinoma; BEAM, branched expression analysis modeling; ICIs, immune checkpoint inhibitors; iCAFs, inflammatory CAFs; irAEs, immune-related adverse events; LR, ligand–receptor; mCAFs, matrix CAFs; MMT, mesenchymal–mesenchymal transition; SCENIC, single-cell regulatory network inference and clustering; scRNA-seq, single-cell RNA-sequencing; TME, tumor microenvironment; TAMs, tumor-associated macrophages; TANs, tumor-associated neutrophils; Tregs, regulatory T cells; TFs, transcription factors; t-SNE, T-distributed stochastic neighbor embedding; vCAFs, vascular CAFs; TMB, tumor mutation burden; TLR, toll-like receptor; TCGA, The Cancer Genome Atlas.
