# Supplementary figures and images for: Dissecting Immunosuppressive Cell Communication Patterns Reveals JunB Proto-Oncogene (JUNB) Shaping a Non-Inflamed Tumor Microenvironment
Source: Front Genet. 2022 Jun 24;13:883583. doi: 10.3389/fgene.2022.883583 (PMC9263213; doi:10.3389/fgene.2022.883583)

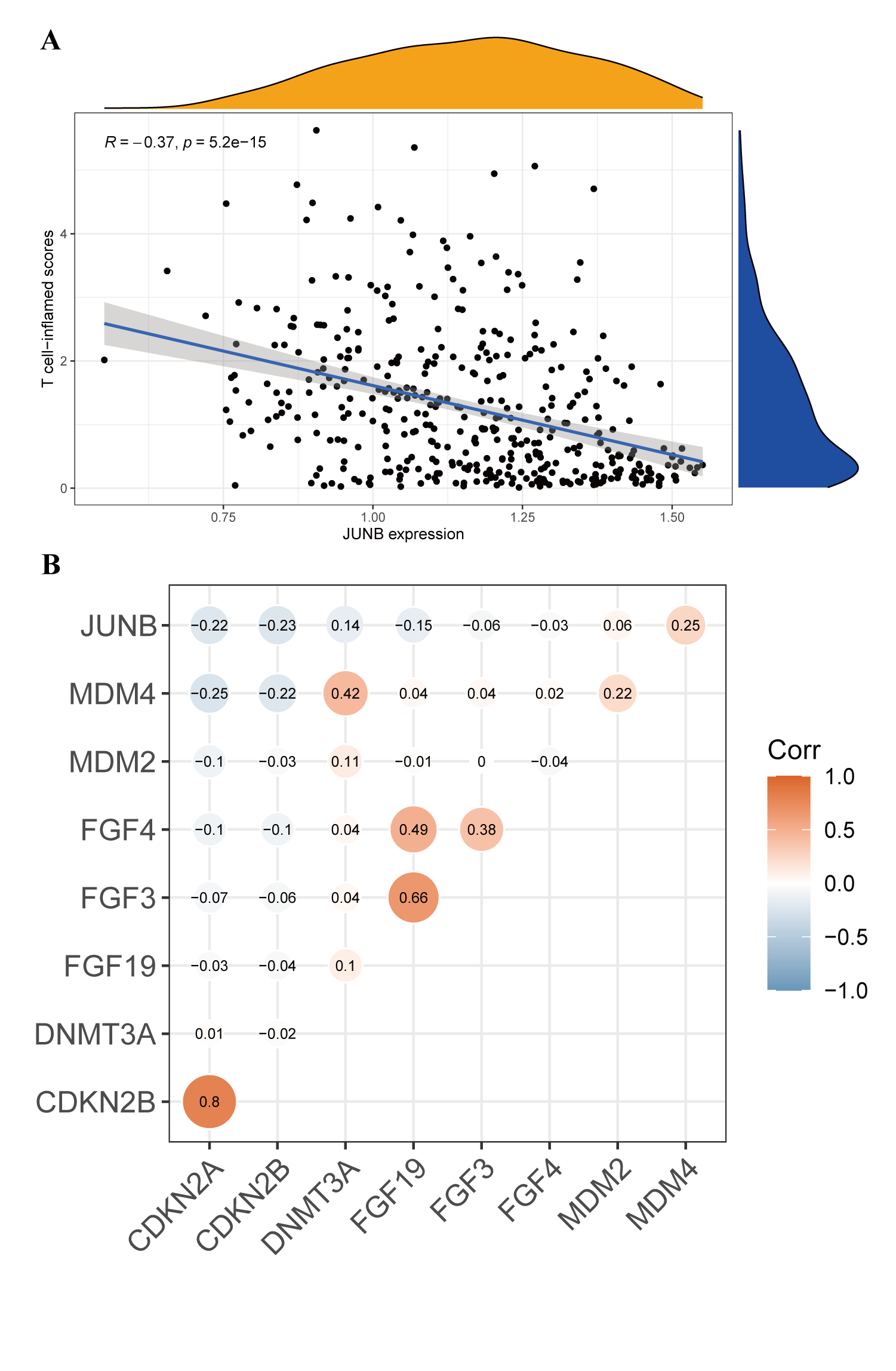

Supplement: Supplementary file 1 [file Image2.TIF]

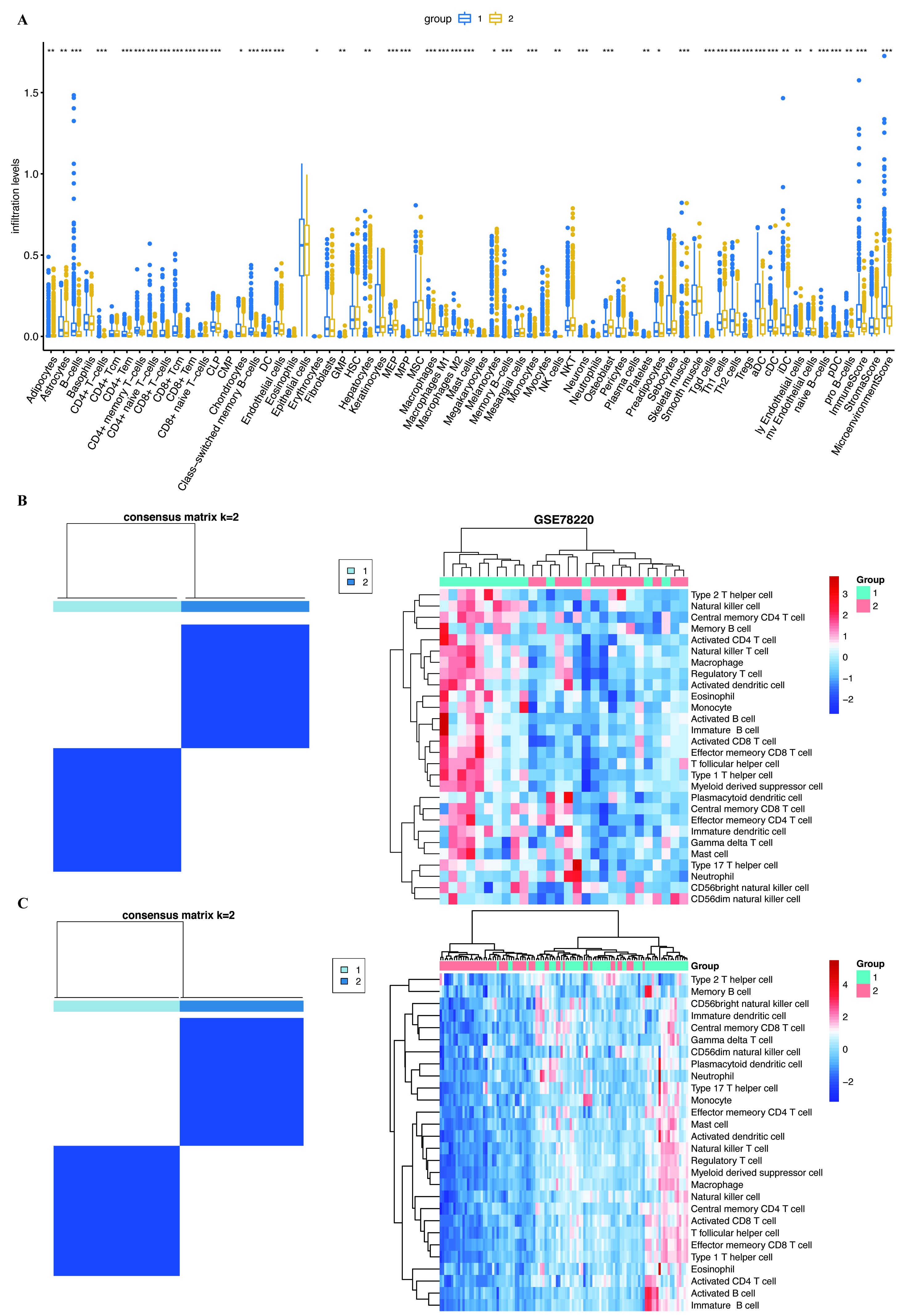

Supplement: Supplementary file 2 [file Image1.TIF]
